# Segmentation of the Striatum from MR Brain Images to Calculate the ^99m^Tc-TRODAT-1 Binding Ratio in SPECT Images

**DOI:** 10.1155/2013/593175

**Published:** 2013-06-18

**Authors:** Ching-Fen Jiang, Chiung-Chih Chang, Shu-Hua Huang, Chia-Hsiang Wu

**Affiliations:** ^1^Department of Biomedical Engineering, I-Shou University, Kaohsiung 82445, Taiwan; ^2^Department of Neurology, Chang Gung Memorial Hospital, Kaohsiung Medical Center, Chang Gung University College of Medicine, Kaohsiung 83301, Taiwan; ^3^Department of Nuclear Medicine, Chang Gung Memorial Hospital, Kaohsiung Medical Center, Chang Gung University College of Medicine, Kaohsiung 83301, Taiwan

## Abstract

Quantification of regional ^99m^Tc-TRODAT-1 binding ratio in the striatum regions in SPECT images is essential for differential diagnosis between Alzheimer's and Parkinson's diseases. Defining the region of the striatum in the SPECT image is the first step toward success in the quantification of the TRODAT-1 binding ratio. However, because SPECT images reveal insufficient information regarding the anatomical structure of the brain, correct delineation of the striatum directly from the SPECT image is almost impossible. We present a method integrating the active contour model and the hybrid registration technique to extract regions from MR T1-weighted images and map them into the corresponding SPECT images. Results from three normal subjects suggest that the segmentation accuracy using the proposed method was compatible with the expert decision but has a higher efficiency and reproducibility than manual delineation. The binding ratio derived by this method correlated well (*R* ^2^ = 0.76) with those values calculated by commercial software, suggesting the feasibility of the proposed method.

## 1. Introduction

Alzheimer's and Parkinson's diseases are two common neurodegenerative diseases associated with the aging process. The induced intellectual and functional deterioration of patients with these diseases can not only bring a heavy load to his/her family but also has an economic impact on society. Early diagnosis with appropriate treatment within a reasonable time frame can prevent abrupt degeneration of these diseases and distressing symptoms. 

The current trend in the early diagnosis of such diseases is usually to adopt a combination of functional images and structural images to inspect the functional and structural changes in specific brain regions. However, qualitative observation alone limits early detection of neurodegenerative diseases, because the associated functional/structural changes are slowly progressive in the early stage and can be too subtle to be detected by human vision. Therefore, quantification of these changes can facilitate early detection of neurodegenerative diseases.

SPECT imaging of dopamine transporter with ^99m^Tc-TRODAT-1 (TRODAT-1) has been proposed to be a valuable and feasible means for the diagnosis of Parkinson's disease and dementia with Lewy bodies (DLB) [1–5]. The specific tracer, TRODAT-1, a radiolabeled tropane that binds dopamine transporters, allows *in vivo* assessment of the presynaptic dopaminergic neuron activity inside the striatum [3, 6]. SPECT images from patients with these diseases reveal a decrease in specific striatal uptake of TRODAT-1 in terms of a dull contrast of radioactivity between the striatum and adjacent brain tissue due to a selective loss of dopamine in the striatum. Even though several approaches show the feasibility of using TRODAT-1 SPECT in the evaluation of patients in the early stages of these neurodegenerative diseases, visual inspection or semi-auto quantification cannot avoid high intra- or interobserver variability and thus hampers the associated diagnostic accuracy [7, 8]. A reliable automatic method could considerably speed up the procedure and make it more reproducible.

Even though some commercialized software packages provide automatic calculation of the TRODAT-1 binding ratio (BR), definition of the striatum in the SPECT image still relies on manual delineation. However, the brain structure is poorly-defined in SPECT images, which reveal more functional information than anatomical structural information. Therefore, demarcation of the region of interest (ROI) in the SPECT image is usually carried out by overlapping the SPECT images with the corresponding MR images, such that physicians can map the ROI delineated in the MR images to the SPECT images. Within this process, there are two key components of determining the accuracy of the TRODAT-1 binding quantification. First, the striatum should be correctly defined. Second, the MR images must be precisely registered with the corresponding SPECT images. However, even a well-trained physician can hardly guarantee obtaining accurate and repeatable results at these two stages. Therefore, this study aims to develop a robust method to fulfill the shortages in the current approaches.

Regarding the segmentation task for subcortical brain structures, several semiautomatic methods have been proposed. Worth et al. proposed the regional thresholding method to segment the caudate from the adjacent tissue [9]. A box was manually located to cover these three tissues, including the ventricles, the caudate, and some white matter, to derive a bimodal histogram, and then the threshold was determined as the mean of the two peaks of the histogram. However, the box location required human determination, and the vague boundaries of the caudate tail surrounded by gray matter still require manual drawing. Barra and Boire proposed a fuzzy-logic-based method to segment subcortical brain structures in MR images by integrating the numerical information derived from the wavelet features and structural information containing symbolized distance and relative direction coding [10]. More recently, Xia et al. took advantage of the high-contrast lateral ventricle as the reference to localize the upper and lower bonds of the caudate nucleus for region growing [11]. Fine-tuning according to the topological and morphological information was still required to smooth the initial segmentation. In view of these methods, as several factors, such as the complex anatomic brain structure, the connection of different tissues of a similar intensity, the heterogeneous intensity within the same class of tissue, and the partial volume effect, limit the performance of fully automatic segmentation of the striatum; therefore, using expert knowledge to refine the initial ROI derived by running the computer program was inevitable. However, visual confirmation and manual correction are conducted slice by slice and thus may still be labor intensive and time consuming. 

Instead of applying expert knowledge in the last step to refine the segmentation in the previous studies, we propose a new approach using an active contour model to reverse the process of segmentation, that is, to let the expert determine the rough location of the striatum and allow the computer to perform the refinement, such that human intervention can be minimized and the segmentation efficiency can be enhanced. The segmented regions were then mapped into the corresponding SPECT images via a hybrid registration method for BR calculation. These methods associated with the imaging protocol are described in detail in Section 2. To verify the reliability of the proposed method, the segmentation results and the derivative BRs were compared with those of experts assisted by commercial software. The results are presented and discussed, following which a brief conclusion is made.

## 2. Methods

**Figure 1:**
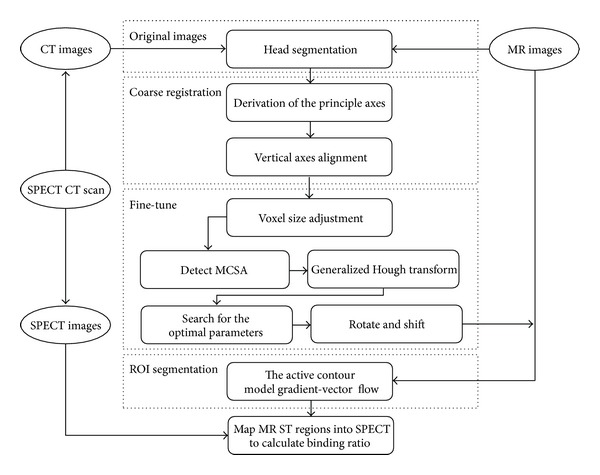
The overall process to derive the BRs in SPECT images via registration of the images from SPECT-CT and MR with ROI segmentation from the registered MR images.

We used hybrid SPECT/CT and 3D T1-weighted images to achieve the goal. Each volume played a distinct role in the overall process. The registration of the MR and the SPECT volume pairs was first conducted using the corresponding CT volume as a medium. After that, the striatum was segmented from the registered MR images. Once the MR images were adjusted to the same size under the same coordinates with the SPECT images through registration, the ROIs obtained by applying the active contour model to the registered MR images could be directly mapped into the SPECT images to calculate the binding potential. The overall process is summarized in Figure 1 and described in detail below.

### 2.1. Imaging Protocol

For this examination, all the patients were injected intravenously with a single bolus dose of 740 MBq (20 mCi) of ^99m^Tc-TRODAT-1. Brain SPECT/CT (Symbia T; Siemens, Erlangen, Germany) images were obtained 4 hours later. The SPECT/CT scanner was equipped with low-energy high-resolution collimators and a dual-slice spiral CT. Acquisition parameters for SPECT were a 128 × 128 matrix, 500 mm FOV with 60 frames (40 s/frame). The scan parameters for the CT were 130 kV, 17 mAs, 5 mm slices, and image reconstruction with a medium-smooth kernel. The SPECT images were attenuation-corrected based on the CT images and scatter-corrected with Flash 3DR algorithm (ordered subsets expectation and 3D maximization with resolution correction) with 8 subsets and 8 iterations.

MR images were acquired using a 3.0 T MRI scanner (Excite, GE Medical Systems, Milwaukee, WI, USA). Structural images were acquired for an anatomical reference using a T1-weighted, inversion-recovery-prepared, three-dimensional, spoiled, and gradient-recalled acquisition in a steady-state sequence with repetition time/inversion time = 8,600 ms/450 ms, a 240 × 240 mm field of view and a 1 mm slice thickness.

### 2.2. Image Registration

To precisely map the ROI delineated from the MR into a corresponding position in the SPECT image, registration of the MR volume with the SPECT-CT volume was required. Even though several automatic registration methods have been proposed, their success is only guaranteed when the two scanning data to be registered contain consistent volumes. However, the clinical volume sets from different image modalities are usually truncated unevenly, lending additional difficulties to the application of conventional registration methods, such as principal axes registration (PAR) or mutual information (MI). To alleviate this problem, we developed a hybrid registration method combining principal axes registration with the general Hough transform [12]. In addition, we took advantage of SPECT-CT, which can acquire SPECT and CT images simultaneously, while the patient maintains his/her position on the same couch. Registration used the CT image as the registration medium to increase the registration accuracy between the SPECT and the MR image volumes. The registration process is fully automatic. The essential idea of the design is briefly described below.

The voxel size was adjusted to a 1 mm^3^ cube through bicubic interpolation prior to the following registration process. The 3D head was segmented as an entity to derive its three principal axes prior to registration. In this two-stage registration scheme, principal axes registration was first applied for coarse registration followed by fine-tuning via applying the general Hough transform to the contour of the maximal cross-sectional area (MCSA). The original concept of principal axes registration (PAR) is to superimpose the two volumes by aligning the corresponding three principal axes from both head volumes [13]. However, the registration accuracy of PAR is restricted by the degree of correspondence between the two sets of principal axes [14]. As the scanning range of one image modality is usually not the same as the other, the centroids of the two different volume sets would not be identical. In consequence, the two sets of principal axes derived from the different centroids do not coincide with each other. Therefore, in the coarse-registration stage, we only adopted PAR to adjust the orientations of the long axis of the head to be parallel with the *z*-axis of the system coordinates. After this stage, the long axis from both head volumes coincided with each other, but the horizontal planes with the two short axes from the two volumes were still mismatched.

In the second stage, the registration error in the horizontal plane was then fine-tuned. We then turned the 3D registration task into a 2D one by searching for the slices containing the (MCSA) in both volumes, in that we had proved the reliability of using the MCSA as the anatomical feature for registration [15]. The vertical shift was first corrected by aligning these two slices; then the detected contour of the MCSA was used to derive the registration parameters via the generalized Hough transform (GHT). The process of the GHT algorithm in this approach included two steps. First, an *R*-table was built by calculating the vector set, $\left\{\;{\overset{⃑}{a}}_{i}\right\}$, between each contour point (*x*_*i*_,  *y*_*i*_) and the center of the contour, *P*_*c*_ (*x*_*c*_,  *y*_*c*_), in the CT image. Then, the corresponding center point (*P*_*c*_′) was derived by searching for the maximal intersection via remapping the vector information to each contour point **X**_*i*_  (*x*_*i*_,  *y*_*i*_) in the MR image. In this study, as there was no scale for reference and the voxel size had been adjusted to be the same, we adapted a robust search only for the rotation angle *β* in (1), when the optimal match between *P*_*c*_ and *P*_*c*_′ was achieved

(1) $$\begin{array}{c} x_{c}=x_{i}+\gamma \cos\left(\theta +\beta \right),\\ y_{c}=y_{i}+\gamma \sin\left(\theta +\beta \right), \end{array}$$

where *θ* is the angle between the directional vector ${\overset{⃑}{a}}_{i}$ and the positive direction of the *x*-axis and *γ* is the length of the ${\overset{⃑}{a}}_{i}$.

The registration parameters of the rigid transform derived above were then applied to the MR volumes to match with the SPECT images. 

### 2.3. ROI Segmentation from MR T1-Weighted Images

**Figure 2:**
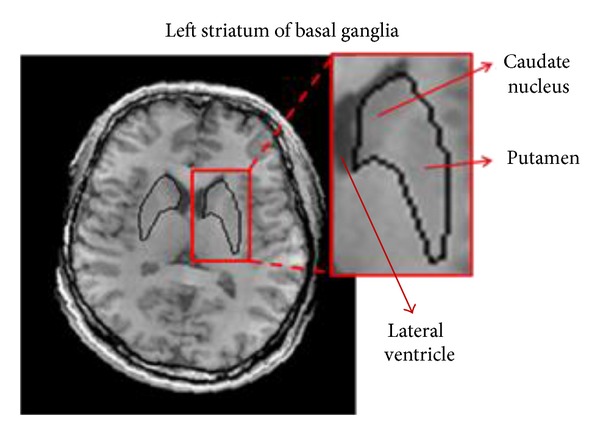
Anatomical structure of the striatum from the axial view of an MR T1-weighted image.

The registered MR T1-weighted images obtained from the previous stage were then used as the reference to demarcate the striatum on the corresponding SPECT images. As the assessment of TRODAT-1 BR is usually carried out from the axial view of SPECT images, the segmentation of the striatum was performed in the axial planes of the MR images.  Figure 2 shows the structure of the striatum from the axial view of the MR image. It can be seen that the left and right sides of striatum of the basal ganglia are located beside the ventricle. Each side of the striatum can be further divided into the caudate nucleus and putamen. We named the two pairs of caudate nucleus and putamen the ST regions. However, the division of the ST regions is not obvious because they usually fuse with other brain structures. The unclear cut between the caudate nucleus and putamen and the surrounding brain structure brings up difficulties in isolating the ST regions solely using automatic image segmentation techniques without any expert intervention. 

To segment these four ROIs, we adopted a modified active contour model. In this way, an initial contour of the first slice can be determined by an expert according to the topological and morphological characteristics of the ST. Once the location and shape of the ST regions are confined into the bond of the initial contour, then refinement can be carried out by the computer according to the intensity information. In addition, assuming smooth variation of the 3D ST region contour, the final contour of the present slice can be directly used as the initial contour for the next slice. To achieve this goal, the active contour model is a suitable choice.

The basis of the active contour model, named snake, is to represent an initial contour in the parametric form of *v*(*s*) = [*x*(*s*), *y*(*s*)], *s* ∈ [0,1] that deforms to the optimal shape by minimizing the energy functional

(2) Esnake=∫01[Eint⁡(v(s))+Eext(v(s))]ds=∫0112[α|v′(s)|2+β|v′′(s)|2+Eext(v(s))]ds,

where *α* and *β* are the parameters to weight the influence on the curve deformation from the curve's tension *v*′(*s*) and the rigidity *v*′′(*s*), respectively. 

Theoretically, at the minima of the energy functional, the snake must satisfy the Euler equation

(3) αv′′(s)−βv′′′′(s)−∇E(v(s))ext=0.

As the first derivative of energy gives the force, the above equation can be interpreted as a force balance equation

(4) $$\begin{array}{c} F_{\mathrm{int}}\left(v\right)+F_{\text{ext}}\left(v\right)=0. \end{array}$$

The internal force, *F*_int⁡_(*v*) = *αv*′′(*s*) − *βv*′′′′(*s*), restricts the curve to stretch and bend, while the external force, *F*_ext_(*v*) = −∇*E*_ext_(*v*), pulls the curve toward the desired image edges. 

The snake is an active rather than a salient model due to the dynamic deformation process by treating the force balance equation as function of time *t*. Therefore, the solution of (3) can be approximated by iteratively searching for the steady state of the following equation, where the *v*(*s*, *t*) = [*x*(*s*, *t*), *y*(*s*, *t*)] denotes *v*(*s*) at the *t*th iteration

(5) ∂v(s,t)∂t=αv′′(s,t)−βv′′′′(s,t)−∇E(v(s,t)).ext

In practice, a numerical solution to (5) can be achieved by discretizing *s* iteratively using a finite difference method [16], as per

(6) xt=(A+γI)−1(xt−1−px,t−1),yt=(A+γI)−1(yt−1−py,t−1),

where **A** is a pentadiagonal matrix containing the constants *α* and *β*. The parameter of *γ* is the step size to control the degree of the contour deformation between iterations. **I** is the unit matrix. **x**_*t*_ and **y**_*t*_ are the vectors consisting of the *x*- and *y*-coordinates of the contour *v*(*s*, *t*), respectively. **p**_*x*,*t*−1_ and **p**_*y*,*t*−1_ are the vectors containing ∂*E*_ext_(*x*(*s*, *t* − 1), *y*(*s*, *t* − 1))/∂*x*, and ∂*E*_ext_(*x*(*s*, *t* − 1), *y*(*s*, *t* − 1))/∂*y* as their elements for all *s*, respectively.

The external force (∇*E*_ext_) in the active model can usually be classified into two types: static and dynamic. Static forces are derived from the image gradients, which do not change throughout the deformation process, while dynamic forces vary as the snake deforms. Using the image gradient as the external force makes the conventional snake difficult to move into a concave edge, because the null image gradients within a homogenous region inside the contour fail to attract the contour, and as a result, the contour is only affected by the internal forces. Even though several dynamic external forces have been proposed to alleviate such a limitation of the static external forces, they also raised other problems, increasing the calculation complexity or leading to uncontrollable deformation [17, 18]. A new static external force, called *gradient*-*vector flow* (GVF), adding the directional property into the original image gradient map, was proposed by Xu and Prince to improve the performance of the static snake in concave edge detection [19]. Several reports have demonstrated the success of applying the GVF snake to medical image segmentation [20–23], including brain MRI [24]. This encouraged us to apply the GVF to segment the ST regions in our study.

The gradient-vector-flow field is defined as **v**(*x*, *y*) = [*u*(*x*, *y*), *v*(*x*, *y*)] such that the external energy function becomes

(7) $$\begin{array}{c} E_{\text{gvf}}=\iint \left\lfloor \mu \left({\left|\nabla u\right|}^{2}+{\left|\nabla v\right|}^{2}\right)+{\left|\nabla f\right|}^{2}{\left|v-\nabla f\right|}^{2}\right\rfloor dx\;dy, \end{array}$$

where *μ* is a parameter to control the degree of smoothness of the gradient-vector-flow field and ∇*f* is an edge map derived from the original image *f*(*x*, *y*).

To solve the equation numerically by discretization and iteration, let *n* indicate the times of iteration, and the increments in *x*, *y*, and *t* are all equal to 1. The relation of vector flows from the current to the next position can be derived as

(8) un+1(x,y)=(1−|∇f|2)un(x,y)+μ[un(x+1,y)+un(x,y+1)    +un(x−1,y)+un(x,y−1)    −4un(x,y)]+|∇f|fx(x,y),vn+1(x,y)=(1−|∇f|2)vn(x,y)+μ[vn(x+1,y)+v(x,y+1)  n    +v(x−1,y)  n+vn(x,y−1)    −4v(x,y)  n]+|∇f|fy(x,y).

There are 4 parameters determined empirically to obtain the optimal results in the approach. The elasticity parameter (*α*) and the rigidity parameter (*β*) in (2) were set to be 0.1 and 0.2, respectively. The parameter (*γ*) in (6) was set to be 1. The external force weight (*μ*) in (7) was set to be 0.5. 

## 3. Results and Discussion

### 3.1. Registration Results

**Figure 3:**
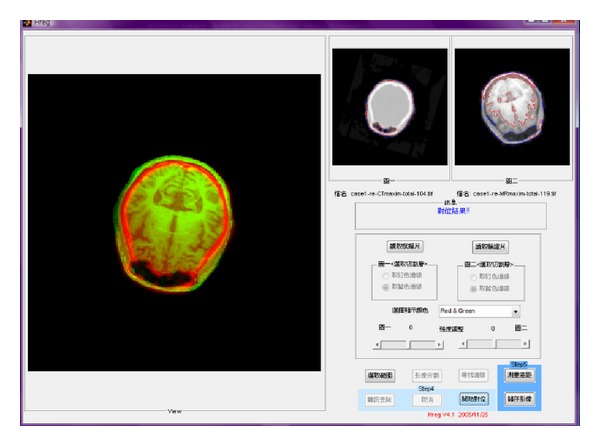
An example of registration of the CT image (middle small panel) and the MR image (right small panel) to render the final fused image (left large panel). The red and blue lines in the small panels are the detected boundaries and the contour of the head.

**Figure 4:**
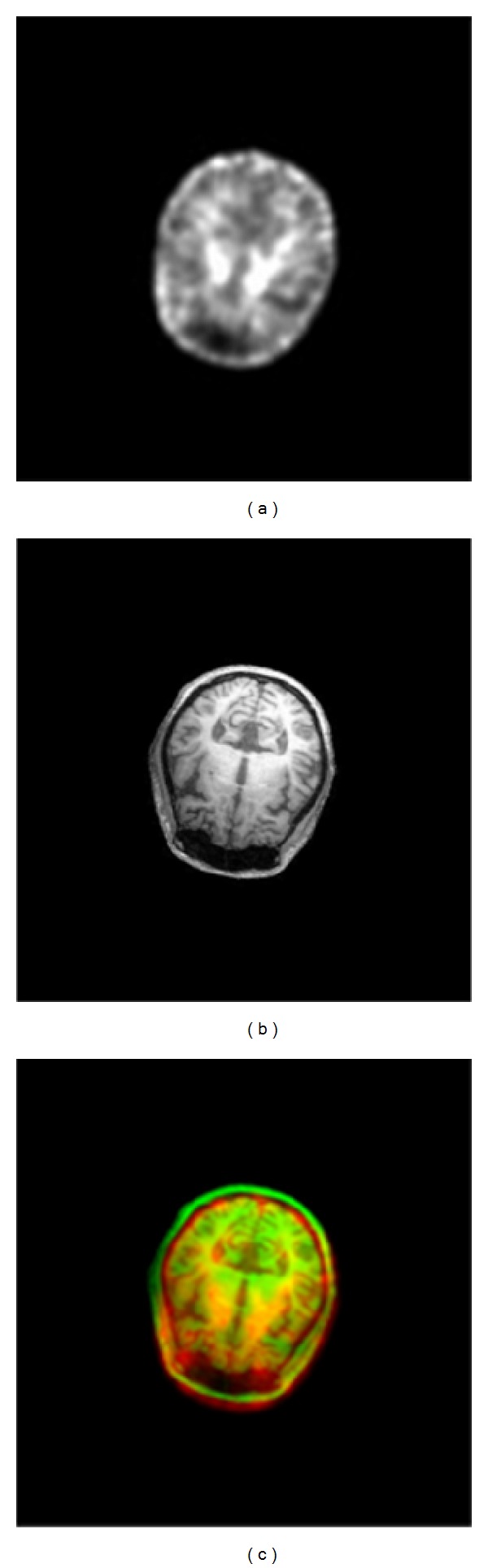
Registration between (a) SPECT and (b) MR to give the final overlaid image in (c).

An example is given in Figure 3 to illustrate the use of our developed interface to detect the slices containing the MCSA from the CT and MR volumes and register these two images through the GHT. Once the rigid transform with the registration parameters had been applied to the MR image volume, it can directly match the SPECT volume, as shown in Figure 4. The registration accuracy reached 96.48%. Our previous study quantitatively evaluated the registration accuracy of the proposed method better than the results obtained solely using the PAR method or directly registering SPECT with MR images [12]. 

### 3.2. Segmentation Results

**Figure 5:**
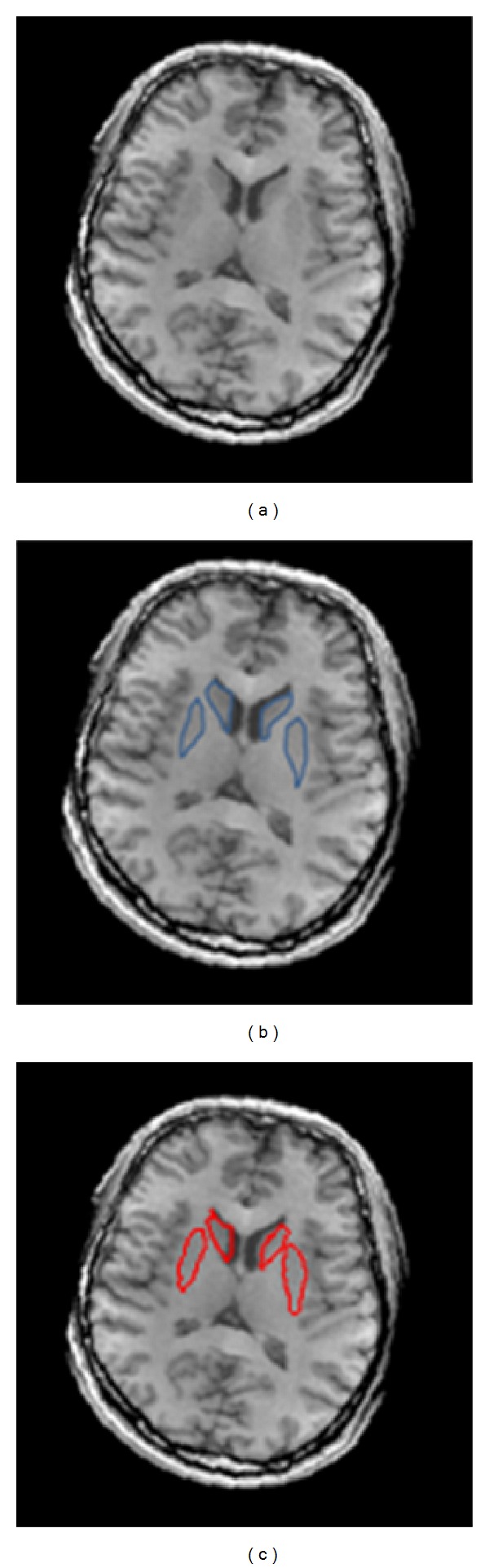
The ST regions in (a) the original MR T1-weighted image and the corresponding segmentation results by (b) manual delineation and (c) the GVF snake.

The expert delineation and the GVF segmentation of the ST regions containing two pairs of the caudate nucleus and putamen are given in Figure 5. A quantitative comparison of these two methods is given below.

We used the Jaccard index (JI) to quantify the degree of match between the two corresponding ROIs. The JI is defined as the ratio of the intersection of two volumes *Ω*_1_ and *Ω*_2_ by the union of them. If the two volumes completely overlap, the JI value is equal to 100%

(9) $$\begin{array}{c} \text{JI}=\frac{\left|\Omega_{1}\cap \Omega_{2}\right|}{\left|\Omega_{1}\cup \Omega_{2}\right|}\times 100\%. \end{array}$$

The five sequential axial slices containing the ST from three normal cases were recruited in the comparative evaluation. Three neurologists first manually delineated the ST regions, including the caudate nucleus and putamen, on two lateral sides of the brain. The intrarater correspondences in terms of the mean and standard deviation of the JI values from the five slices are listed in Table 1, suggesting great differences between observers. It was found that Raters A and C had the highest correspondence, with a JI value greater than 70%.

The JI values were also derived by mapping the manually defined contours by each rater into the GVF segmented results. The initial contour of the first slice in each case was defined by the same specialist in the GVF snake process.  Table 2 shows the correspondence with GVF segmentation. We used the paired *t*-test to evaluate the significance level between the JIs derived from the interrater comparison and those from the rater-GVF comparison for each slice in each case. The insignificant differences (*P* = 0.124 under a 95% confidence interval) suggest that the segmentation accuracy using the GVF snake is compatible with the level of manual drawing.

Rater A, the chief neurologist, was required to conduct the manual drawing twice. The JI values of the two delineations are listed in the middle column of Table 3, showing that the correspondence declined along with the slice number. Instead of segmentation solely by hand, the GVF snake was also applied twice to the same set of images. Only the first slice required an initial contour manually defined by the rater each time. The JI values of repeated conduction of the GVF snake are also listed in the third column of Table 3, suggesting more stable results than those from slice-by-slice manual drawing. 

In comparison with the index of overlap (similar to JI) between hand-drawing and computer-aided segmentation reported in the literature [9–11], the JI values obtained in our study were relatively low. This could be due to the extra region, that is, the putamen, involved in our study. The segmentation target of the previous reports is focused on the caudate nucleuses that are next to ventricles with greater contrast (Figure 1) and therefore more easily identified. In comparison with the caudate nucleuses, the low contrast of the putamen to the surrounding tissue increases the difficulty of extraction. Using expert hand drawing as the comparison basis seems to be the only choice in current studies, since there is no gold standard to determine the absolute accuracy of segmentation of the ST regions due to individual-dependent variation in the brain structure. However, we demonstrated that significant interobserver and intraobserver variability in such a decision exists even among the well-trained neurologists participating in our study, which was overlooked in previous studies. The inconsistency in decision-making could be incurred by the small size (in the order of 100 pixels) of the structures as compared with the imaging resolution and image noise. Under a compatible level of precision as shown in Tables 1 and 2, we demonstrated that the reproducibility and consistency improved when using the GVF snake segmentation method. In addition to stability, the GVF snake can save labor and provide a more efficient way than previous studies to define the ST regions contours in consecutive slices, as it only requires an initial contour drawn by hand in the first slice. 

### 3.3. Binding Ratio Calculation

**Figure 6:**
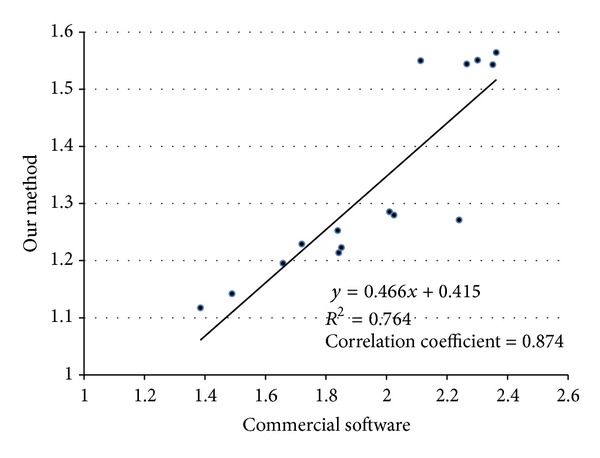
Linear regression analysis between the BRs derived by our method and those by the commercial software.

In the final stage of evaluation of the reliability of the proposed method, after completing the MR and SPECT image registration, the BRs were also derived from the segmented ST regions in the SPECT images using the proposed method to compare with those obtained using commercial software (Siemens Medical Systems, Knoxville, TN, USA), in which the ST regions were manually outlined by an expert. The BR was calculated by normalizing the mean intensity in the ST regions by the mean intensity in the occipital cortices. Linear regression analysis (Figure 6) revealed a close correlation (CC = 0.874, under 95% confidence interval) between the BRs derived by the two systems.

## 4. Conclusions

To calculate the regional TRODAT-1 binding ratio in SPECT studies, accurate and repeatable extraction of the ST regions from MR images is required to indirectly define the corresponding regions in the SPECT images. Segmentation directly on the SPECT image is not applicable in this case, because it distorts the ST regions. Clinical routine tends to apply manual delineation of the ST regions, which is prone to errors incurred through interobserver and intraobserver variability. Previous researchers have developed several segmentation algorithms to complete similar tasks, where expert decisions for anatomical and morphological information were still necessary to refine the results. As the localization of the ST regions is a knowledge-driven task, the proposed method allowed the expert to assign the initial contour in the proper location and applied the gradient-vector-flow snake to approach the real contours. In such a way, the complexity of the algorithm can be reduced and the efficiency of segmentation can be increased. Results from three normal subjects showed a higher reproducibility of the proposed method than manual segmentation under compatible segmentation accuracy. The MR images with segmented ST regions were overlaid on the SPECT images using our previously developed registration algorithm to calculate the TRODAT-1 BR. The derived BRs correlated well with those derived using commercial software, suggesting a good reliability of the proposed method. 

## Figures and Tables

**Table 1:** Correspondence of the manual delineation between the observers.

JI (%)	Rater A-Rater B	Rater B-Rater C	Rater A-Rater C
Case 1	60.4 ± 4.8	62.1 ± 1.1	75.6 ± 2.1
Case 2	54.3 ± 4.5	54.7 ± 5.6	71.8 ± 5.2
Case 3	58.0 ± 4.7	58.1 ± 5.4	76.6 ± 5.2

**Table 2:** Correspondence between manual delineation and the GVF snake result for each observer.

JI (%)	Rater A-GVF snake	Rater B-GVF snake	Rater C-GVF snake
Case 1	64.4 ± 9.0	56.2 ± 5.5	65.3 ± 4.8
Case 2	68.6 ± 1.6	57.7 ± 5.1	65.4 ± 6.5
Case 3	61.1 ± 3.8	51.5 ± 3.1	59.9 ± 3.7

**Table 3:** Correspondence between two repeated conductions of each method.

Slice no.	JI (%)	
	Manual drawing	GVF deformation
1	66.3	77.05
2	61.2	73.31
3	53.34	78.3
4	51.3	71.46
5	45.57	78.44
Mean ± std	55.5 ± 8.2	75.7 ± 3.2

std: standard deviation.
